# Nationwide real-world data on tumor-agnostic genomic companion diagnostics and comprehensive genomic profiling tests

**DOI:** 10.3892/mco.2026.2981

**Published:** 2026-10-16

**Authors:** Yasushi Yatabe, Katsutoshi Oda, Hiroya Taniguchi, Kuniko Sunami, Russell Miller, Shotaro Yasuoka, Ryosuke Watanabe

**Affiliations:** 1 Department of Diagnostic Pathology, National Cancer Center Hospital, Tokyo 104-0045, Japan; 2 Department of Obstetrics and Gynecology, The University of Tokyo, Tokyo 113-0033, Japan; 3 Department of Clinical Oncology, Aichi Cancer Center Hospital, Nagoya, Aichi 464-8681, Japan; 4 Department of Laboratory Medicine, National Cancer Center Hospital, Tokyo 104-0045, Japan; 5 Syneos Health Japan K.K., Tokyo 100-7012, Japan; 6 Medical Affairs, MSD K.K., Tokyo 102-8667, Japan; 7 BeOne Medicines G.K., Minato, Tokyo 105-7114, Japan

**Keywords:** cancer, comprehensive genomic profiling, genomic medicine, biomarker testing, companion diagnostics

## Abstract

The present study aimed to assess the utilization of biomarker testing, including companion diagnostics and comprehensive genomic profiling (CGP), in Japan and to highlight the value of large-scale real-world database analysis in precision oncology. Claims data from the Medical Data Vision Co., Ltd. database, covering ~40 million patients across 26% of advanced treatment hospitals in Japan, were retrospectively analyzed. Biomarker tests for five genomic biomarkers (BRAF, BRCA1/2, KRAS/NRAS, homologous recombination deficiency and microsatellite instability (MSI)) were evaluated in patients with a confirmed diagnosis of one of 11 common cancer types, a cancer of unknown primary origin (CUP) or a rare cancer, who underwent at least one biomarker test between December 2018 and March 2022. A total of 41,191 biomarker tests were recorded in 28,382 patients, with test volumes increasing markedly between 2018 and 2021. MSI testing was the most common (70.1% of patients), followed by RASKET-B for KRAS/NRAS (25.5%) and BRAF (23.9%). Patients with colorectal cancer frequently underwent multiple tests (42.8%) and distinct testing sequences were observed in gynecological cancer. CGP was more commonly performed on tissue-based samples than through liquid biopsy, particularly in rare cancer (38.5%) and CUP (11.4%). Notably, ~40% of patients received a new treatment after CGP, whereas only 1.7% initiated indicated therapy after MSI testing. The mean time to subsequent treatment initiation from the index date was 70.4 days. In conclusion, the present nationwide real-world study provides an early comprehensive overview of the prevalence and trends of biomarker testing in Japan, and reveals a gap between testing and treatment implementation. Although testing is increasing, its effective clinical use remains limited. Large-scale database analyses may help identify barriers and support strategies to improve the integration of biomarker testing into treatment decision-making, ultimately advancing precision oncology.

## Introduction

Biomarker testing has revolutionized cancer care by enabling more precise and personalized treatment strategies (1). Within this landscape, comprehensive genomic profiling (CGP) and companion diagnostics (CDx) serve distinct yet complementary roles. CGP is a broad genomic testing approach used in cancer genomic medicine to detect molecular alterations that may be clinically relevant across multiple cancer types, even if targeted therapies are not yet available. In contrast, CDx tests identify specific biomarkers that determine patient eligibility for approved targeted therapies.

Beyond genomic testing, biomarker assessment also includes methods such as immunohistochemistry (IHC) and general blood-based biomarkers (e.g., tumor markers), which provide additional insights into cancer biology. Since the approval of genomic panel testing in Japan in 2019 as part of Japan's Cancer Genomic Medicine program, biomarker-based precision treatment has become increasingly accessible to patients (2). However, because CGP is still relatively new in Japan and several CDx tests have only recently become eligible for reimbursement under the national health insurance system, real-world data on the utilization of biomarker testing remain limited.

According to Japanese cancer statistics, approximately one million new cancer cases were diagnosed in 2019. The most common cancer types were colorectal, lung, and stomach cancer, with prostate cancer being the most common among males and breast cancer among females (3). Biomarker testing, particularly CDx and CGP, plays a critical role in optimizing treatment for these prevalent cancers (4). CDx tests enable clinicians to select targeted therapies by identifying biomarkers that predict a patient's response to treatment, thereby helping to optimize the risk-benefit profile of the selected therapy (5). In contrast, CGP enables the simultaneous analysis of multiple genes and molecular alterations, thereby providing a comprehensive genomic overview of a tumor (6,7). By identifying potential therapeutic targets and improving understanding of tumor heterogeneity, CGP serves as a valuable tool in advancing precision oncology.

Tumor-agnostic approaches, which classify tumors based on molecular and genomic characteristics rather than tissue of origin, represent an emerging area of precision oncology. Biomarkers such as microsatellite instability-high (MSI-H), NTRK fusions, and high tumor mutational burden (TMB) are used to guide treatment selection across multiple cancer types.

Numerous biomarker tests have been approved for various targets since the first CDx was approved in Japan in 2011(8). Additional CDx platforms are now used in clinical practice and research in Japan for specific cancer types, including BRACAnalysis CDx^®^ and myChoice^®^ CDx (9), OncoBEAM™ RAS Colorectal Cancer (CRC) kit (10), RASKET™-B kit (11) and the Microsatellite Instability *In Vitro* Diagnostic (MSI-IVD) kit (12). This study aimed to provide a comprehensive overview of biomarker testing practices across cancer types prevalent in Japan, thereby providing insights into the implementation of precision medicine and personalized treatment approaches in the Japanese population.

## Patients and methods

### Data source

This study used claims data from a hospital-based database maintained by Medical Data Vision Co., Ltd. (MDV) to examine the real-world utilization of CDx and CGP. This database contains anonymized hospital-based health insurance claims data from approximately 40 million patients in Japan (13). It includes inpatient and outpatient medical care data from approximately 26% of the 440 advanced treatment hospitals across Japan. Medical claims data are collected from all insurance types. Approximately 35% of the data are derived from patients aged 65 years and above (13). The study cohort consisted of 28,382 patients (15,035 males and 13,347 females). The median age was 69 years, with an age range of 18-99 years. The database allows for longitudinal tracking of patients over time within the same institution. Patient information includes demographics, clinical diagnoses (International Classification of Diseases, 10th Revision; ICD-10), procedures and prescriptions.

### Study design

This study was a retrospective cohort study assessing the frequency and utilization of biomarker tests in Japan. Patients were identified from the MDV database using specified ICD-10 diagnosis codes for cancer types of interest and claims for biomarker tests of interests, enabling a comprehensive overview of biomarker testing practices (definitions in Table SI). The study period was from 01 December 2017 to 30 June 2022 (see Fig. S1). Index date was defined as the date of the first claim for any pre-specified biomarker within the identification period, spanning from 01 December 2018 (coinciding with approval of the first biomarker test of interest, the MSI-IVD kit) to 31 March 2022. To ensure sufficient follow-up for the identification of treatments initiated after biomarker testing, patients who underwent CDx testing were required to have at least 3 months of follow-up after index date, whereas patients who underwent CGP testing were required to have 8 months of follow-up.

### Study population

Eligible patients were required to have a confirmed diagnosis (utagaiflg=0, indicating a confirmed diagnosis record in the MDV database) of a cancer type of interest and at least one claim for a relevant biomarker test during the identification period. Patients aged <18 years and those without at least two claims within 12-month look-back period or the required follow-up period (≥3 months for CDx and ≥8 months for CGP) were excluded.

### Data analysis

Key variables included the frequency of CDx and CGP utilization, the types of biomarkers tested, and cancer types for which these tests were performed. Five genomic biomarkers: BRAF, BRCA1/2, KRAS/NRAS, homologous recombination deficiency (HRD) and MSI, were selected based on their availability in the database, their relevance for patient stratification and treatment decisions, and their utility for broader comparative analysis across cancer types. To enhance clarity, the analysis focused on genomic biomarker tests because many IHC-based biomarkers, including programmed death-ligand 1 (PD-L1), are approved as complementary diagnostics. These tests may not directly influence clinical decision-making in the same manner as CDx. Accordingly, IHC-based tests were excluded. CGP was included because it is reimbursed under the national health insurance system and is used to guide treatment decisions in clinical practice, similar to CDx. Since biomarker-driven clinical practice has been relatively well-established in lung and breast cancers, biomarker tests for these cancer types were excluded from this study.

Newly initiated treatment was defined as any claim for chemotherapy, targeted therapy or an immune checkpoint inhibitor (ICI) that did not occur during the 12-month look-back period prior to index date, and that was recorded after index date through the end of the study period. For patients with a BRCA1/2 or HRD test for ovarian cancer on index date, treatment initiation post-biomarker testing was defined as any claim for newly initiated treatment that was not recorded during the 12-month period prior to index date, and that was recorded after index date through the end of the study period. Data were analyzed using descriptive statistics, including frequencies, the prevalence and utilization of CDx and CGP in different cancer types. The primary objective was to describe real-world biomarker testing utilization, testing sequences, temporal changes, and treatment initiation patterns; therefore, the analyses were descriptive rather than inferential.

## Results

### Characteristics of the study population and overall utilization of biomarker testing

A total of 28,382 patients had a confirmed cancer diagnosis and underwent at least one biomarker test (Fig. 1). Overall, 59.3% (16,819/28,382) of patients had a metastatic solid tumor, and 98.4% (27,926/28,382) having any malignancy including lymphoma and leukemia. The mean follow-up time was 349.8 days (SD: 263.9). A total of 41,191 tests were recorded from index to end of follow-up.

The cohort had a mean age of 67.5 years (SD: 11.3) and 53% were male. Mean body weight at index date was 56.9 kg (SD: 13.2) (Table SII). The mean modified Charlson comorbidity index score was 5.3 (SD: 2.4). Common comorbidities were mild liver disease (22.6%, 6,400/28,382) and chronic pulmonary disease (13.7%, 3,889/28,382; Table SII).

Overall biomarker testing frequency increased from 7,198 claims for tests in 2019 to 8,878 claims in 2020 and 10,088 claims in 2021 (full years available in the dataset).

### Differences among biomarker tests

The most common claims for biomarker tests by overall patient count were for CDx, including MSI (70.1%, 19,894/28,382) and RASKET-B for KRAS/NRAS (25.5%, 7,232/28,382) and BRAF (23.9%, 6,793/28,382). BRCA1/2 testing for prostate cancer was the least common CDx claim (0.4%, 106/28,382). Finally, 1,557 patients (5.5%) underwent tissue-based CGP, and 35 (0.1%) had liquid biopsy CGP (Table SII).

Of the 21,065 MSI tests that were conducted during the study period, 53.8% (11,323 tests) were performed in patients with colorectal cancer, while 19.3% (4,074 tests) were for those with rare cancers (data not shown).

### Temporal trends in MSI testing utilization

After regulatory approval of MSI testing, the total monthly number of claims was relatively consistent across tumor types and indicated treatment (Figs. 1 and 2). This pattern was relatively consistent across tumor types and indicated treatment. MSI testing utilization appeared to correspond with the timing of approvals of specific ICIs, including pembrolizumab and nivolumab, for specific indications. For example, increases in MSI testing were observed among patients with gastric and esophageal cancer following approval of these therapies for the respective indications (Fig. 2). Despite the increasing utilization of MSI testing over time, only 1.7% (342/19,894) of patients subsequently initiated pembrolizumab or nivolumab following MSI testing. The mean time from MSI testing to treatment initiation was 38.8 days (SD: 22.3) (Table I).

### Frequency and utilization of biomarker testing by cancer type

Overall, 22.2% (7,892/35,582) of patients underwent multiple biomarker tests on the index date. This was most common among patients with colorectal cancer (42.8%; mean 1.6 tests per patient), followed by prostate cancer (11.5%; 107/932) and rare cancers (11.0%; 490/4,471; Table II). During follow-up, 4.7% of patients (1,681/35,582) had at least one additional biomarker test.

Testing sequences varied by cancer type (Table II). For example, among gynecological cancers, approximately 90% of patients with cervical cancer (565/647) and endometrial cancer (1,246/1,344) patients received MSI testing only, while fewer than 10% underwent tissue-based CGP only, the second most common testing pattern. An even smaller proportion of patients (<3.5%) underwent MSI testing on index date followed by tissue-based CGP during follow-up.

For patients with ovarian cancer, the testing pattern differed notably. BRCA1/2 testing only was observed in 40.9% (900/2,202), whereas MSI testing only was observed in 39.7% (875/2,202) of cases. In addition, 12.7% (279/2,202) of patients underwent HRD testing only, and 4.0% (88/2,202) underwent tissue-based CGP only (the fourth most common testing sequence, data not shown). BRCA1/2 testing was used as the second and third most common biomarker tests among patients with pancreatic cancer (477/3,260; 14.6%) and prostate cancer (105/932; 11.3%), respectively, whereas in ovarian cancer it was the most common testing pattern (900/2,202; 40.9%).

Among patients with ovarian cancer, a total of 913 BRCA1/2 (other cancers) tests and 295 HRD tests were performed at index date (Table III). BRCA1/2 testing was introduced in May 2019, whereas HRD testing did not begin until January 2021. Following regulatory approvals related to BRCA1/2 and HRD testing, MSI testing in ovarian cancer declined, whereas BRCA1/2 and HRD testing became more common (Fig. 3). Following BRCA1/2 testing, 23.0% (210/913) of patients initiated olaparib, a poly(ADP-ribose) polymerase inhibitor (PARPi), with a mean time from testing to treatment initiation of 220.4 days (SD: 213.8). However, only a small proportion of patients with prostate cancer and pancreatic cancer received olaparib as a new treatment during the follow-up period (Table I). After BRCA1/2 testing, only 3.8% (4/106) of patients with prostate cancer and 2.5% (13/518) of those with pancreatic cancer received new treatment before the end of their claims record in the database. Targeted therapies were the most common treatment among patients with prostate cancer who received treatment after CGP (37.9%; 58/153), whereas chemotherapy was most common treatment among patients with pancreatic cancer (41.2%; 98/238). The mean time from testing to treatment initiation with olaparib was 36.5 days (SD: 25.7) for prostate cancer and 51.2 days (SD: 23.9) for pancreatic cancer.

Approximately 40% of patients across cancer types received new treatment after CGP. Among patients underwent tissue-based CGP, 7.1% (110/1,557) subsequently initiated ICI therapy. The mean time from CGP testing to initiation of treatment was 86.5 days (SD: 65.6) overall, and 96.2 days (SD: 65.3) for patients who initiated ICI therapy (Table IV). When stratified by cancer type, 60% of patients with cancer of unknown primary origin (CUP) or rare cancers did not receive treatment after tissue-based CGP. Patients with CUP most frequently received chemotherapy (29/177; 16.4%), whereas those with rare cancers most frequently initiated targeted therapy (123/600; 20.5%). A plain language summary of these findings is available as Data S1.

## Discussion

This study provides a unique nationwide, multi-cancer perspective on biomarker testing patterns within a single healthcare system in Japan, addressing limitations of previous studies focusing on specific tumor types or geographic regions. Although testing levels quickly plateaued within the first few years after approval, the initial increase, particularly in MSI and BRCA1/2 testing, is consistent with global trends in oncology practice and epidemiology (14,15). For example, the National Comprehensive Cancer Network (NCCN) guidelines recommend MSI testing for all patients with a history of colorectal cancer (16). Recent real-world studies from the United States and Spain report relatively high MSI testing rates and increasing utilization over time (17,18), reflecting improved adherence to guideline recommendations, although variability across settings remains. These findings are consistent with our results, which demonstrated increased MSI testing following regulatory approval in Japan. Notably, our study extends prior evidence by providing a nationwide, multi-cancer overview of biomarker testing patterns and addressing gaps in existing country-specific and tumor-specific analyses.

Emerging insights from large genomic datasets reveal differences between ethnicities, such as White and Asian populations, in genomic alteration patterns, including the frequency of cancer driver mutations (19), and real-world evidence indicates that MSI testing uptake also varies by tumor type. Although MSI testing is relatively well established in colorectal cancer, earlier studies in endometrial cancer reported lower testing rates (approximately one-third in Europe), with more recent data demonstrating increasing uptake over time (20,21). In Japan, treatment guidelines for genomic medicine primarily focus on other biomarkers but also recommend MSI testing to guide decisions regarding further treatment with ICI (22). The observed differences in MSI testing utilization likely reflect variations in clinical practice and disease characteristics rather than superiority of testing in any specific cancer (20,21). These findings suggest that discrepancies in testing and clinical application may be driven by a combination of factors, including disease-specific characteristics, regulatory timelines, and practical barriers in clinical adoption. Because the MDV database did not include individual biomarker test results, these discrepancies should be interpreted as differences in test use and post-testing treatment initiation, rather than biomarker-positive detection rates. Furthermore, although biomarker testing plays an important role in treatment selection, patients in routine clinical practice often present with more heterogeneous characteristics than those in clinical trials. Consequently, even when biomarker results are positive, they may not necessarily lead to the use of the corresponding recommended therapies in routine care.

The predominance of MSI testing in colorectal cancer patients and the high frequency of multiple tests in this group suggest a more integrated role of MSI tests in managing colorectal cancer. This pattern may reflect the specific molecular characteristics of colorectal cancer, which require assessment of a broader range of biomarkers (including KRAS/BRAF) for effective treatment stratification (23). The relationship between MSI-H and BRAF mutations in colorectal cancer is crucial for predicting clinical characteristics and prognosis, as described in the Japanese treatment guidelines (24). However, since this study was based on claims data, biomarker test results were unavailable, including the percentage of MSI-H and BRAF-mutated cancers. Future research should further investigate this relationship and its clinical implications.

Differences in MSI testing utilization across cancer types may reflect variation in clinical guidelines and the prevalence of individual cancer types. Akagi *et al* (25) tested 26,237 clinical samples of multiple cancer types and reported a relatively high MSI-H prevalence in several cancers with historically limited treatment options, including endometrial (16.9%), colorectal (3.8%), gastric (6.7%), esophageal (1.1%), as well as ovarian (2.2%) and pancreatic (0.8%) cancers. In the current nationwide analysis, testing rates were high in gastric cancer (97.3%) and endometrial cancer (98.2%), but lower in ovarian cancer (47.1%). However, patients in this study were required to have at least one claim for any of these five biomarkers. Therefore, the reported testing rates reflect the distribution of biomarker testing among tested patients rather than among all patients diagnosed with a given cancer type. This distinction should be considered when interpreting the results.

During the study period, Japanese treatment guidelines recommended MSI testing before initiating second-line treatment, particularly for gastric cancer (26), based on the effectiveness of pembrolizumab in MSI-H tumors. Similarly, Japanese treatment guidelines for endometrial cancer also recognize the effectiveness of pembrolizumab for MSI-H cases (27). In contrast, ovarian cancer guidelines only make a cautious reference to pembrolizumab use in patients with MSI-H tumors because of insufficient supporting data (28). These differences reflect a more selective and evidence-based application of genomic testing across cancer-specific treatment strategies.

The relatively stable mean time to treatment initiation post-MSI testing across tumor types suggests a relatively uniform response to testing results in routine clinical practice in Japan. However, the low proportion of patients who received a new treatment after testing indicates potential barriers to translating test results into therapeutic action (29). Detection of MSI-H is important for identifying tumors that may respond to pembrolizumab in gastric cancer and potentially other ICIs (30). Furthermore, emerging real-world evidence suggests that not only testing itself but also the time from biomarker testing to treatment initiation is critical, as delays in testing or subsequent therapy initiation may be associated with poorer survival outcomes (31). The relatively low proportion of patients who newly initiated treatment after biomarker testing may have several explanations, including negative or non-actionable test results, prior use of relevant therapy before the index date, clinical deterioration before treatment initiation, limited treatment access, or transfer to another institution following testing, resulting in subsequent therapies not being captured in the database. Because claims data do not include reasons for clinical decision-making, these factors could not be separately evaluated.

BRCA1/2 testing plays an important role not only in detecting germline mutations but also in guiding treatment decisions. The introduction of BRCA1/2 testing for hereditary breast and ovarian cancer (HBOC) syndrome has been a key factor driving its utilization. Following approval of BRCA analysis as a CDx for olaparib treatment for BRCA1/2 mutated ovarian cancer, the number of BRCA1/2 tests increased from 2019 onward (32). Consistent with this observation, real-world evidence demonstrates a marked increase in BRCA testing uptake over time, with the proportion of patients tested within one year of diagnosis increasing substantially across successive years (33).

However, the adoption of BRCA1/2 testing declined following approval of HRD tests as CDx for the PAOLA-1 regimen at the end of 2020(34). Real-world evidence suggests that, despite widespread BRCA testing, the uptake of HRD testing remains relatively low, with substantially fewer patients undergoing HRD testing compared with BRCA testing in clinical practice. This pattern may reflect the later approval and gradual adoption of HRD testing, as well as practical considerations such as testing complexity, and suggests that HRD testing is likely to increase over time in a manner similar to the earlier expansion of BRCA testing following its clinical adoption (35). It indicates that the landscape of biomarker testing in clinical practice is substantially influenced by the availability of specific tests and the approval of associated therapies.

One real-world study in Japan found that 26.8% of newly diagnosed advanced ovarian cancer patients had tissue BRCA1/2 mutations, with 60.0% having HRD (36). Considering the high prevalence of HRD in ovarian cancer, and the convenience of MyChoice CDx test from Myriad Genetics, HRD testing may become more common than BRCA1/2 testing in current clinical situation (37). The MyChoice CDx test assesses both HRD positivity and tumor BRCA1/2 mutation status; however, it cannot distinguish between germline and somatic BRCA1/2 mutations.

Olaparib was approved in Japan in 2020 as a treatment option for BRCA mutated pancreatic and metastatic castration-resistant prostate cancer (34). Given this relatively recent approval during the study inclusion period, the lower testing rates observed in these cancer types may reflect later adoption of BRCA CDx testing compared with ovarian cancer, where BRCA testing and PARP inhibitor use were established earlier. This relatively low prevalence of BRCA mutations in pancreatic cancer (38) and metastatic castration-resistant prostate cancer (39) may also have contributed to the lower utilization of BRCA CDx testing.

The number tissue-based CGP tests was 45-fold higher than that of liquid biopsy-based CGP during the study period. One factor contributing to this difference is the earlier regulatory approval of tissue-based CGP (2018) compared with liquid biopsy-based CGP (2021) in Japan (40,41). Given the study period, this difference in timing likely had a substantial impact on utilization. Although Japanese clinical oncology practice guidelines authored by the Japanese Cancer Association, the Japanese Society of Medical Oncology, and the Japan Society of Clinical Oncology have recommended the use of liquid biopsy in certain situations (40), several factors may have contributed to its lower use in practice. These include challenges in determining the optimal timing of testing, concerns regarding false-negative results due to limited ctDNA sensitivity, and practical barriers such as lack of familiarity and variability in reimbursement. Although specific reasons could not be evaluated in this descriptive database study, such barriers may nevertheless exist and may have influenced the observed adoption patterns (42,43). These include the limited availability of approved liquid biopsy tests, as well as perceived differences in sensitivity and reliability compared to tissue biopsies. Additionally, ICI treatments were frequently initiated after tissue-based CGP but not after liquid biopsy-based CGP, suggesting a higher level of clinical confidence in tissue-based results for guiding immunotherapy decisions. However, a recent study investigating FoundationOne Liquid testing in Japan between 2021 and 2023 reported increasing utilization over time and suggested that liquid biopsy testing may provide therapeutic benefits in several distinct cancer types (44).

Biomarker-driven treatment selection plays a critical role across multiple cancer types, guiding the use of targeted therapies and immunotherapies. MSI-H testing helps identify patients eligible for treatment with ICI (25,41), while other biomarkers, such as BRAF and KRAS mutations, influence treatment selection by predicting treatment response or resistance. Similarly, BRCA1/2 and HRD testing inform the use of PARP inhibitors, particularly in cancers in which deficiencies in DNA repair influence therapeutic response.

In the context of CUP and rare cancers, the high percentage of patients not receiving treatment after CGP testing warrants attention. The notable low rate of access to treatment following both tissue-based and liquid biopsy-based CGP highlights challenges in identifying actionable targets and the limited availability of testing options for Japanese patients. Limited access to genotype-matched therapy post-CGP is a known issue in Japan (45-47). To address these obstacles, academic institutions are actively working to enhance patient enrollment in clinical studies guided by CGP results. For CGP, this gap may reflect the absence of actionable alterations, limited access to approved genotype-matched therapies or clinical trials, deterioration of patient condition before treatment initiation, and institutional restrictions because CGP is available through designated cancer genomic medicine institutions.

A recent systematic review has highlighted the important role for liquid biopsy in prostate cancer for accurately detecting multiple biomarkers and reducing costly false positives (48). This situation accentuates the need for more research and drug development in these areas.

Limitations of the claims database include the inability to track patients across institutions and the lack of information on biomarker test results, actionable alterations, performance status, disease stage, line of therapy, treatment intent, and reasons for not receiving subsequent treatment. Therefore, subsequent treatments after transfer to another institution may have been underestimated. In addition, differences between CDx and CGP testing may partly reflect institutional resource differences, because CDx tests are more widely available whereas CGP is restricted to designated cancer genomic medicine institutions. These limitations should be considered when interpreting the observed gap between biomarker testing and treatment initiation. Therefore, the present study focused on descriptive analyses of nationwide biomarker testing and treatment initiation patterns.

Although formal statistical comparisons across cancer types or trend analyses over time could technically be performed, we considered such analyses unlikely to provide clinically meaningful insights within the context of this study. The included cancer types differ fundamentally with respect to biomarker indications, clinical practice guidelines, regulatory approval and reimbursement timelines, and available targeted therapies, all of which substantially influence testing practices. As a result, statistical comparisons would largely reflect these inherent differences rather than differences attributable to biomarker utilization itself. Furthermore, because study eligibility criteria required patients to have undergone at least one biomarker test, the reported proportions describe biomarker testing patterns within a selected cohort of tested patients rather than the frequency of biomarker testing among all patients with a given cancer type. Accordingly, inferential comparisons across cancer types or formal trend analyses were considered unlikely to provide additional clinically meaningful insight beyond the descriptive findings presented.

In conclusion, biomarker testing in routine oncology care in Japan is still emerging, driven by rapidly expanding treatment options and growing insights into tumor biology. This nationwide real-world study provides an early comprehensive view of biomarker testing prevalence and utilization trends, revealing a persistent gap between testing and treatment initiation. Although descriptive in nature, this study provides clinically meaningful insights, particularly given the lack of available methodologies at the time of study initiation for comprehensively characterizing real-world testing patterns.

Although biomarker testing is increasing, its effective integration into clinical practice remains limited. Further analyses of large healthcare databases may help identify barriers and support the development of targeted strategies to advance precision oncology, including broader dissemination of guideline-recommended testing and improved integration of biomarker testing into treatment decision-making. These findings provide clinically relevant insights to inform strategies for optimizing the implementation of biomarker-driven treatment in routine practice.

## Supplementary Material

Plain language summary

Study period and index date. CDx, companion diagnostics; CGP, comprehensive genomic profiling.

Diagnosis codes for cancers of interest.

Patient characteristics overall and by biomarker test.

## Figures and Tables

**Figure 1:**
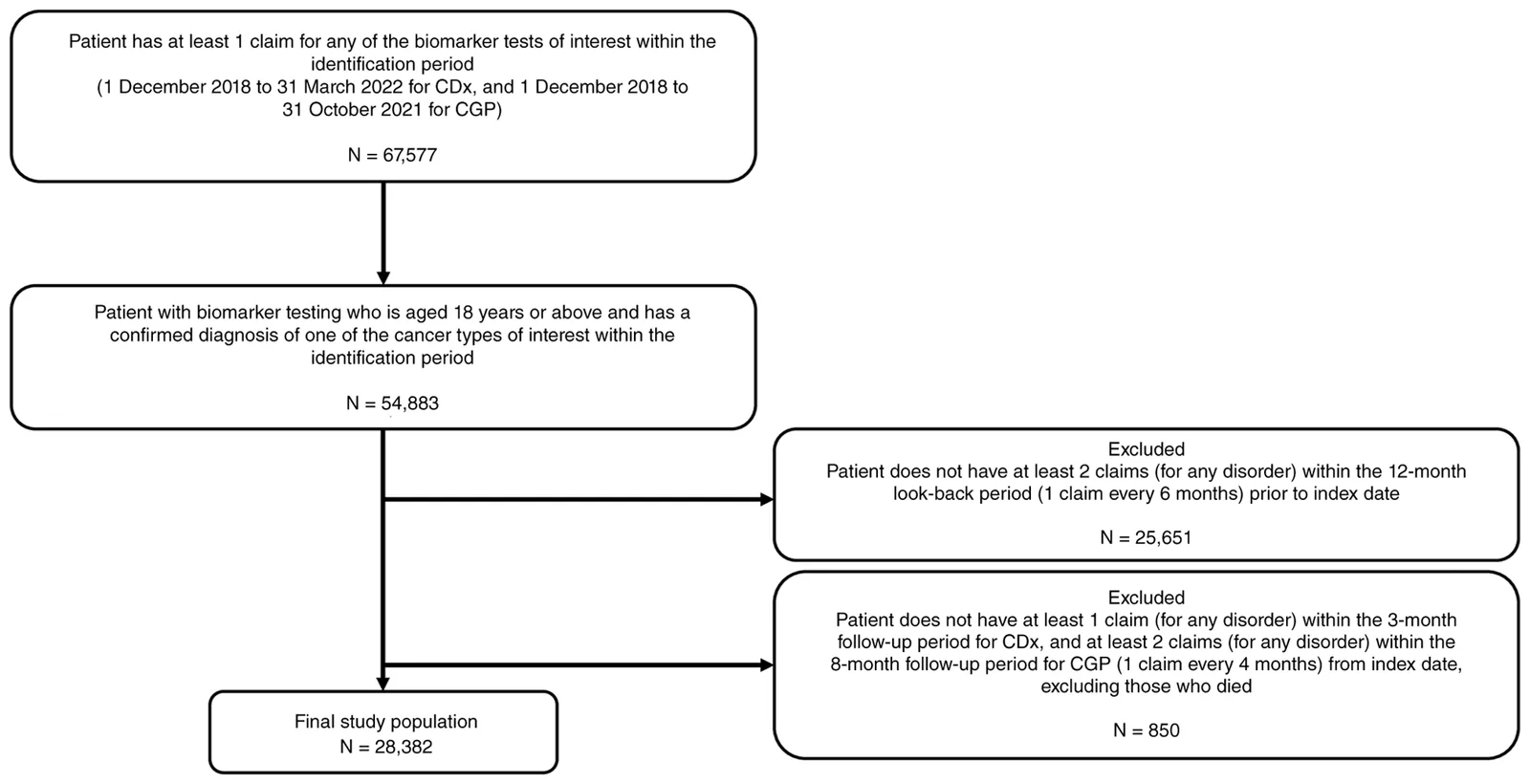
Patient selection flowchart. This schematic flow shows the process of identifying the target patient population in the database. Patients who met the inclusion and exclusion criteria were included in the study population. Patients were identified based on pre-specified biomarker test claims and confirmed cancer diagnoses within the MDV database. CDx, companion diagnostics; CGP, comprehensive genomic profiling; MDV, Medical Data Vision Co., Ltd.

**Figure 2:**
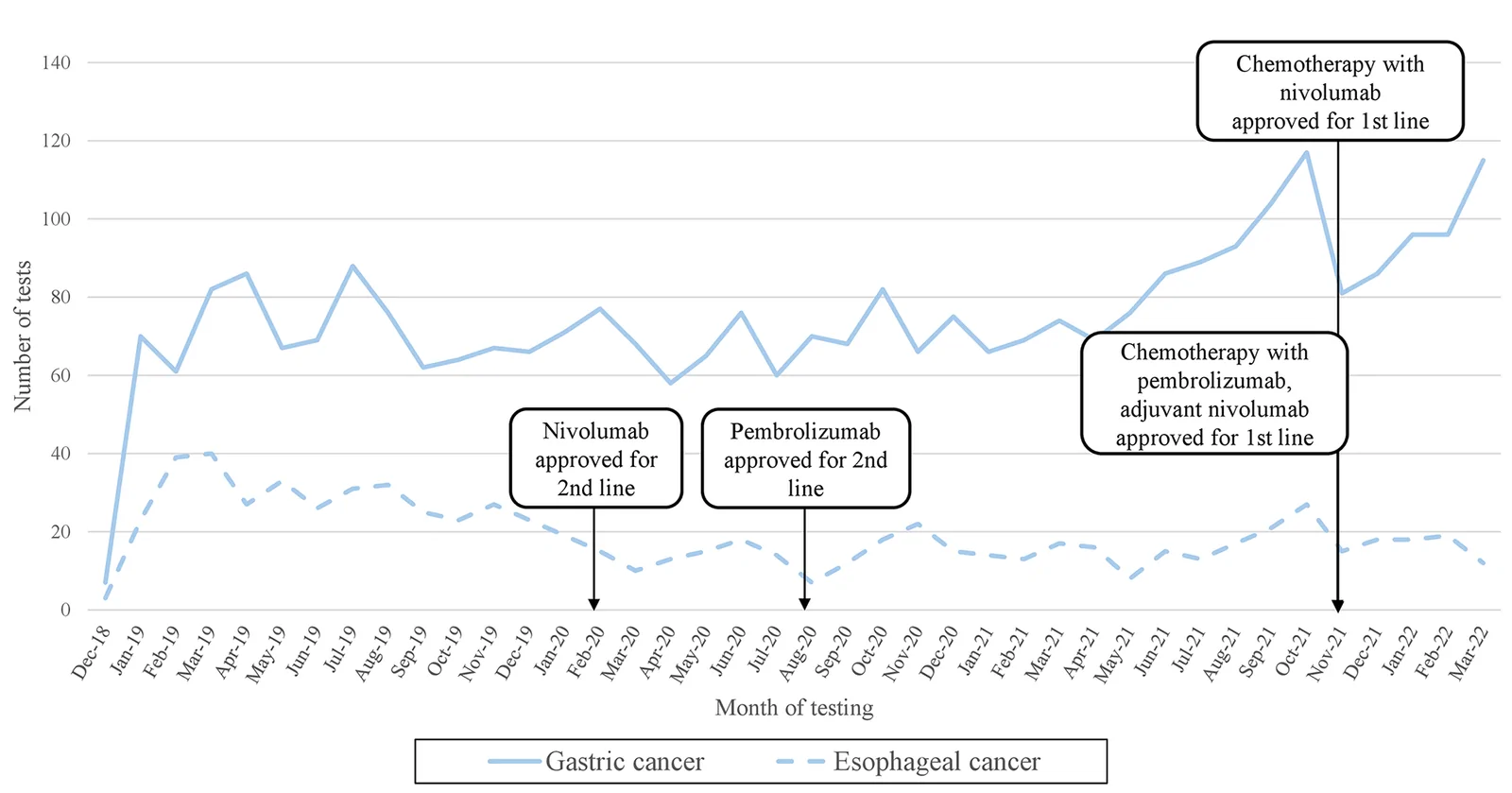
Utilization of MSI testing in gastric and esophageal cancer over time. This figure represents the monthly number of MSI tests conducted for gastric cancer (blue solid line) and esophageal cancer (blue dotted line) between December 2018 and March 2022. The x-axis represents the month of testing and the y-axis the number of tests recorded in the database. MSI, microsatellite instability.

**Figure 3:**
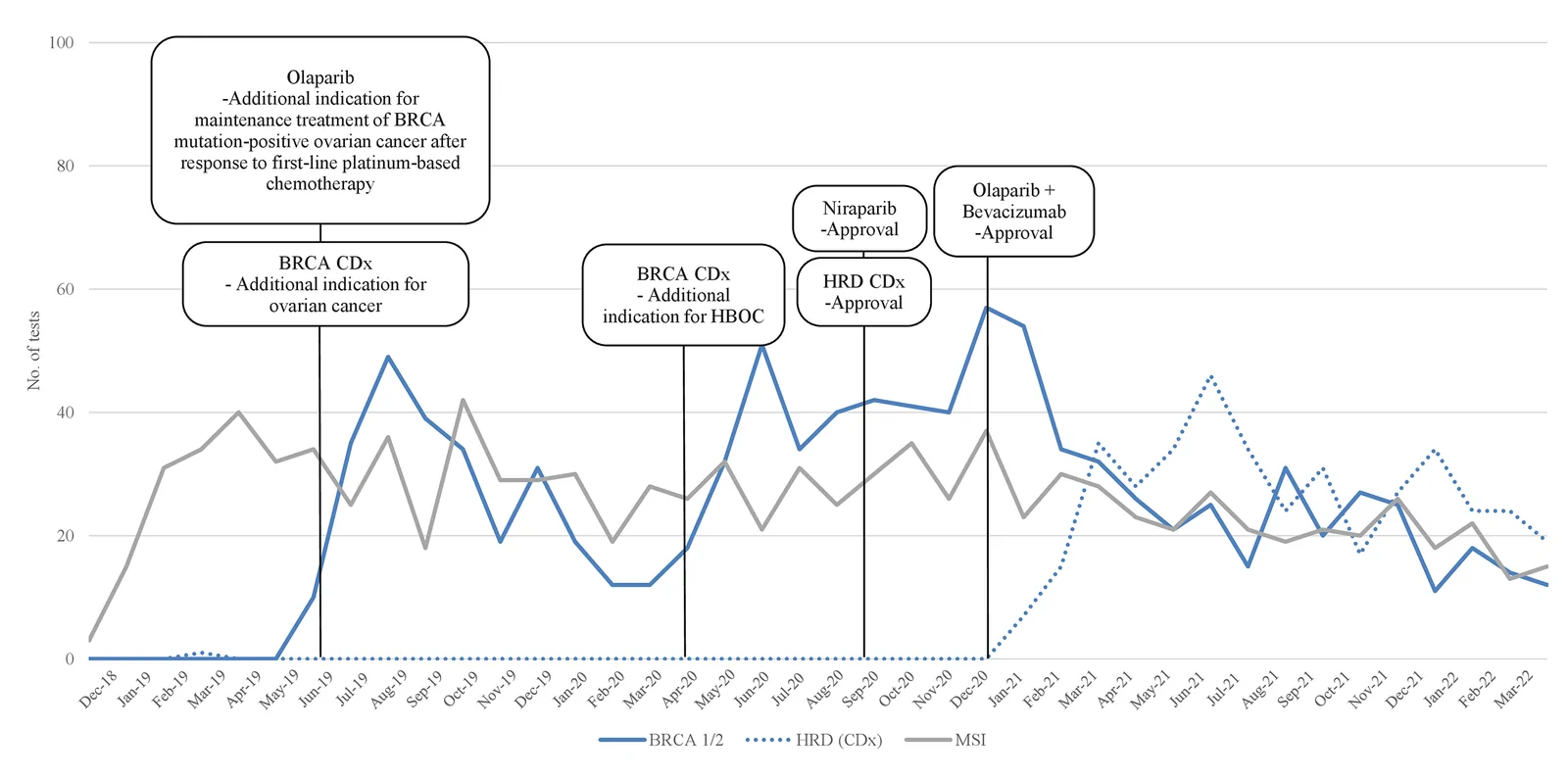
BRCA/HRD/MSI testing in ovarian cancer over time. The monthly number of BRCA1/2 (blue solid line), HRD (blue dotted line) and MSI (gray solid line) tests performed between December 2018 and March 2022 are shown. The x-axis represents the month of testing and the y-axis the number of tests recorded in the database. CDx, companion diagnostics; HBOC, hereditary breast and ovarian cancer; HRD, homologous recombination deficiency; MSI, microsatellite instability.

**Table I:** Time to treatment after biomarker testing at index date; overall cohort, n=28,382.

A, MSI (CDx)-pembrolizumab					
		Patients newly treated with indicated treatment		Time to treatment initiation from index date, days	
Cancer type	Total (n)	n	%	Mean	SD
Total	19,894	342	1.7	38.8	22.3
Gastric cancer	2,936	51	1.7	38.8	24.1
Colorectal cancer (nivolumab)	10,433	34	0.3	31.3	21.0
Colorectal cancer	10,433	121	1.2	36.4	21.9
Pancreatic cancer	2,549	27	1.1	39.9	16.7
Biliary tract cancer	832	14	1.7	34.9	23.7
Prostate cancer	663	15	2.3	46.0	21.5
Cervical cancer	583	10	1.7	38.6	22.7
Endometrial cancer	1,273	98	7.7	42.0	21.7
Ovarian cancer	930	14	1.5	43.7	25.3
Cancer of unknown primary origin	1,668	39	2.3	41.7	24.1
Rare cancer	3,875	86	2.2	39.2	22.9
B, BRCA 1/2 (CDx)-olaparib					
		Patients newly treated with indicated treatment		Time to treatment initiation from index date, days	
Cancer type	Total (n)	n	%	Mean	SD
Prostate cancer	106	4	3.8	36.5	25.7
Other cancers: Total	1,422	223	15.7	210.5	211.3
Ovarian cancer	913	210	23.0	220.4	213.8
Pancreatic cancer	518	13	2.5	51.2	23.9

**Table II:** Biomarker testing patterns, overall and by cancer type.

						Sequence of biomarker testing including index date and follow-up								
		Patients with more than 1 biomarker test on index date		Patients with at least 1 test during follow-up		Sequence 1			Sequence 2			Sequence 3		
Cancer type	Total number of patients	n	%	n	%	Tests	n	%	Tests	n	%	Tests	n	%
Overall (claims)	35,582	7,892	22.2	1,681	4.7									
Gastric cancer	3,103	168	5.4	66	2.1	MSI	2,768	89.2	CGP (Tissue Biopsy)	165	5.3	MSI-H + RASKET-B (BRAF) + RASKET-B (KRAS/NRAS)	143	4.6
Esophageal cancer	837	29	3.5	26	3.1	MSI	728	87.0	CGP (Tissue Biopsy)	78	9.3	MSI + RASKET-B (BRAF) + RASKET-B (KRAS/NRAS)	24	2.9
Colorectal cancer	15,812	6,760	42.8	699	4.4	MSI	7,574	47.9	RASKET-B (BRAF) + RASKET-B (KRAS/NRAS)	3,898	24.7	MSI + RASKET-B (BRAF) + RASKET-B (KRAS/NRAS)	2,376	15.0
Pancreatic cancer	3,260	78	2.4	258	7.9	MSI	2,474	75.9	BRCA (other indications)	477	14.6	CGP (Tissue Biopsy)	226	6.9
Biliary tract cancer	899	13	1.4	38	4.2	MSI	819	91.1	CGP (Tissue Biopsy)	66	7.3	MSI ->CGP (Tissue Biopsy)	26	2.9
Prostate cancer	932	107	11.5	73	7.8	MSI	557	59.8	CGP (Tissue Biopsy)	153	16.4	BRCA (prostate cancer)	105	11.3
Cervical cancer	647	19	2.9	31	4.8	MSI	565	87.3	CGP (Tissue Biopsy)	62	9.6	MSI ->CGP (Tissue Biopsy)	22	3.4
Endometrial cancer	1,344	27	2.0	38	2.8	MSI	1,246	92.7	CGP (Tissue Biopsy)	71	5.3	MSI ->CGP (Tissue Biopsy)	19	1.4
Ovarian cancer	2,202	59	2.7	119	5.4	BRCA (other indications)	900	40.9	MSI	875	39.7	HRD	279	12.7
Lung cancer	232	1	0.4	5	2.2	CGP (tissue biopsy)	227	97.8	CGP (liquid biopsy)	4	1.7	CGP (tissue biopsy) -> CGP (liquid biopsy)	3	1.3
Cancer of unknown primary origin	1,843	141	7.7	86	4.7	MSI	1,528	82.9	CGP (tissue biopsy)	173	9.4	MSI + RASKET-B (BRAF) + RASKET-B (KRAS/NRAS)	91	4.9
Rare cancer	4,471	490	11.0	242	5.4	MSI	3,389	75.8	CGP (tissue biopsy)	581	13.0	MSI + RASKET-B (BRAF) + RASKET-B (KRAS/NRAS)	300	6.7

The denominator for all proportions is the total number of patients for each cancer type; test names connected by ‘+’ represent claims with the same date and those by ‘->’ different dates. CDx, companion diagnostics; CGP, comprehensive genomic profiling; HRD, homologous recombination deficiency; MSI, microsatellite instability.

**Table III:** Number of patients with MSI, BRCA and CGP tests at index, overall and by cancer type.

Cancer type	Number of patients with tests performed	MSI	BRCA 1/2 Prostate Cancer (CDx)	BRCA 1/2 Other Cancers (CDx)	HRD (CDx)	Tissue Biopsy (CGP)
Overall	28,382	19,894	106	1,422	295	1,557
Gastric cancer	3,103	2,936	NA	NA	NA	167
Esophageal cancer	837	757	NA	NA	NA	78
Colorectal cancer	15,812	10,433	NA	NA	NA	207
Pancreatic cancer	3,260	2,549	NA	518	NA	238
Biliary tract cancer	899	832	NA	NA	NA	68
Prostate cancer	932	663	106	NA	NA	153
Cervical cancer	647	583	NA	NA	NA	68
Endometrial cancer	1,344	1,273	NA	NA	NA	75
Ovarian cancer	2,202	930	NA	913	295	92
Lung cancer	232	NA	NA	NA	NA	228
Cancer of unknown primary origin	1,843	1,668	NA	NA	NA	177
Rare cancer	4,471	3,875	NA	NA	NA	600

Dx, companion diagnostics; CGP, comprehensive genomic profiling; MSI, microsatellite instability; NA, not available.

**Table IV:** Time to treatment after CGP testing at index date; overall cohort, n=28,382.

A, Tissue-based CGP						
			Patients newly treated with indicated treatment		Time to treatment initiation from index date, days	
Cancer type	n	Treatment type	n	%	Mean	SD
Total	1,557	Total	637	40.9	86.5	65.6
		Chemotherapy	235	15.1	77.3	65.5
		Targeted therapy	312	20.0	93.5	66.4
		Immune Checkpoint	110	7.1	96.2	65.3
		Inhibitors (ICI)				
Gastric cancer	167	Total	60	35.9	83.5	64.9
		Chemotherapy	18	10.8	86.4	65.9
		Targeted therapy	19	11.4	85.2	67.1
		ICI	28	16.8	95.6	72.3
Esophageal cancer	78	Total	21	26.9	67.0	55.9
		Chemotherapy	4	5.1	74.0	38.8
		Targeted therapy	5	6.4	97.8	68.8
		ICI	12	15.4	51.9	53.5
Colorectal cancer	207	Total	88	42.5	78.4	59.9
		Chemotherapy	15	7.2	71.5	59.9
		Targeted therapy	66	31.9	80.6	61.2
		ICI	9	4.3	89.6	59.4
Pancreatic cancer	238	Total	112	47.1	79.4	62.7
		Chemotherapy	98	41.2	76.8	64.0
		Targeted therapy	10	4.2	109.5	49.8
		ICI	4	1.7	69.8	46.3
Biliary tract cancer	68	Total	23	33.8	61.9	59.2
		Chemotherapy	20	29.4	65.7	62.3
		Targeted therapy	1	1.5	8.0	NA
		ICI	2	2.9	51.0	7.1
Prostate cancer	153	Total	71	46.4	85.0	62.7
		Chemotherapy	10	6.5	74.3	65.0
		Targeted therapy	58	37.9	86.4	64.4
		ICI	5	3.3	108.2	42.4
Cervical cancer	68	Total	27	39.7	77.7	60.2
		Chemotherapy	16	23.5	74.1	62.4
		Targeted therapy	9	13.2	85.9	65.2
		ICI	3	4.4	99.0	55.1
Endometrial cancer	75	Total	36	48.0	105.0	74.0
		Chemotherapy	18	24.0	85.6	64.5
		Targeted therapy	14	18.7	120.9	72.1
		ICI	8	10.7	154.3	82.2
Ovarian cancer	92	Total	28	30.4	89.5	81.3
		Chemotherapy	9	9.8	53.7	72.5
		Targeted therapy	18	19.6	110.1	82.3
		ICI	2	2.2	133.0	128.7
Lung cancer	228	Total	95	41.7	93.2	62.7
		Chemotherapy	21	9.2	99.5	79.3
		Targeted therapy	49	21.5	95.6	62.3
		ICI	29	12.7	96.2	55.0
Cancer of unknown primary origin	177	Total	71	40.1	99.0	60.2
		Chemotherapy	29	16.4	73.2	64.2
		Targeted therapy	23	13.0	121.9	55.4
		Immune Checkpoint Inhibitors	21	11.9	115.8	45.9
Rare cancer	600	Total	245	40.8	90.4	67.2
		Chemotherapy	86	14.3	76.5	64.1
		Targeted therapy	123	20.5	105.2	68.4
		ICI	47	7.8	84.4	67.1
B, Liquid biopsy-based CGP						
			Patients newly treated with indicated treatment		Time to treatment initiation from index date, days	
Cancer type	n	Treatment type	n	%	Mean	SD
Total	35	Total	14	40.0	89.6	63.6
		Chemotherapy	5	14.3	55.8	57.0
		Targeted therapy	9	25.7	108.3	62.0
		ICI	0	0.0	NA	NA

Time period for analysis is entire study period. CDx, companion diagnostics; CGP, comprehensive genomic profiling; ICI, immune checkpoint inhibitors; MSI, microsatellite instability; NA, not available.

## Data Availability

The data generated in the present study are not publicly available due to licensing restrictions but may be requested from the corresponding author.
