# Supplementary material for: Nationwide real-world data on tumor-agnostic genomic companion diagnostics and comprehensive genomic profiling tests
Source: Mol Clin Oncol. 2026 Oct 16;25(6):72. doi: 10.3892/mco.2026.2981 (PMC13725746; doi:10.3892/mco.2026.2981)

Data S1. Plain language summary

Biomarker testing is used to identify changes in DNA, RNA, proteins, and other biological markers found in tumor tissue or in blood. These biomarkers can help doctors better understand a patient's cancer and select treatments that are more likely to be effective. In Japan, the availability of biomarker tests for cancer patients has increased since the introduction of the Cancer Genomic Medicine program.

In this study, we examined how biomarker tests are used in routine cancer care in Japan. We assessed utilization patterns for five biomarkers (BRAF, BRCA1/2, KRAS/NRAS, HRD and MSI) across different cancer types. Using a database of administrative claims for medical procedures, we examined the use of biomarker tests among patients with cancer from the end of 2018 through March 2022.

The use of biomarker testing and its impact on treatment decisions varied across cancer types. Biomarker testing increased markedly between 2018 and 2021, with MSI testing being the most commonly used test. Testing patterns differed even within gynecological cancers. Patients with colorectal cancer frequently underwent multiple tests, whereas patients with many other cancer types typically received only one or two tests.

Comprehensive genomic profiling (CGP) is a type of biomarker testing that evaluates multiple genetic alteration in a single test. In our study, CGP performed on tumor tissue samples were more common than CGP performed using blood samples. In many cancer types, including rare cancers and cancers of unknown primary origin, most patients did not receive a new treatment after biomarker testing. Overall, of the type, frequency, and order of biomarker testing were generally consistent with clinical guidelines. Biomarker testing patterns were strongly influenced by the availability of companion diagnostic tests and approved treatment options.

This analysis of a large health claims database provides valuable insights into how biomarker testing is used in routine clinical practice in Japan. The findings highlight unmet needs and potential strategies to improve precision cancer care in Japan.

Figure S1. Study period and index date. CDx, companion diagnostics; CGP, comprehensive genomic profiling.

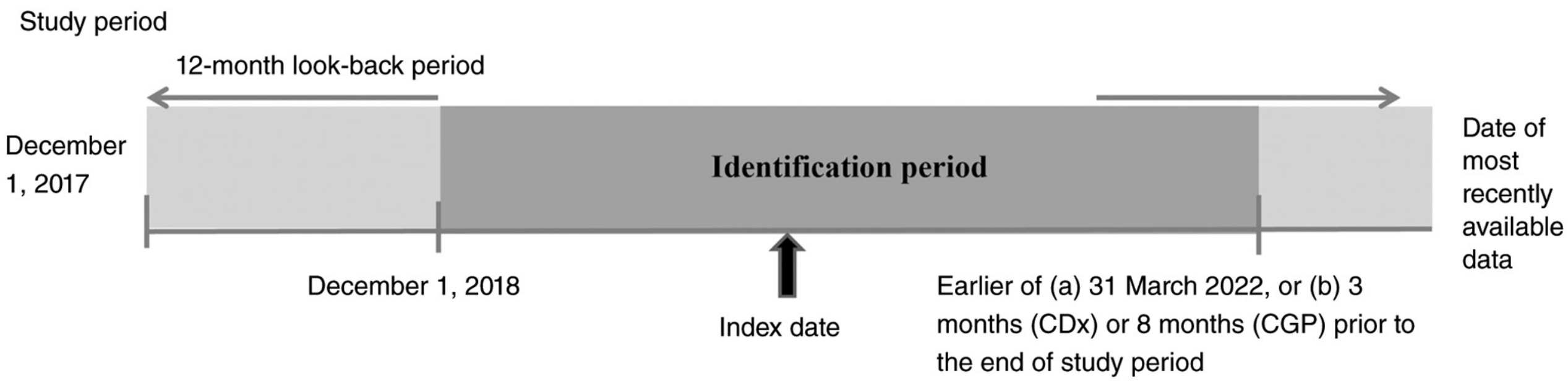

Supplement: Supplementary file 1 [file Supplementary_Data1.pdf]
