# Supplementary material for: Nationwide real-world data on tumor-agnostic genomic companion diagnostics and comprehensive genomic profiling tests
Source: Mol Clin Oncol. 2026 Oct 16;25(6):72. doi: 10.3892/mco.2026.2981 (PMC13725746; doi:10.3892/mco.2026.2981)
Supplement: Supplementary file 2 [file Supplementary_Data2.pdf]

Table SI. Diagnosis codes for cancers of interest.

Cancer type	ICD-10 code
Gastric cancer	C16: C16.0, 16.1, 16.2, 16.3, 16.4, 16.5, 16.6, 16.8, 16.9
Esophageal cancer	C15: C15.0,15.1, 15.2, 15.3, 15.4, 15.5, 15.8, 15.9
Colorectal cancer	C18: C18.0-9, C19, C20
Pancreatic cancer	C25: C25.0, 25.1, 25.2, 25.3, 25.4, 25.7, 25.8, 25.9
Biliary Tract Cancer	C24: C24.0, 24.8, 24.9
Prostate cancer	C61
Cervical cancer	C53: C53.0, 53.1, 53.8, 53.9
Endometrial cancer	C54: C54.0, 54.1, 54.2, 54.3, 54.8, 54.9
Ovarian cancer	C56
Lung cancer	C34x
Cancer of unknown primary origin	C80: C80.0, 80.9
Rare cancers	Conversion of rare cancer ICD-O-3 (source: http://rarecarenet.istitutotumori.mi.it/rarecarenet/index.php/cancerlist) to ICD-10 (https://seer.cancer.gov/tools/conversion/)

ICD-10, International Classification of Diseases, 10th Revision; ICD-O-3, International Classification of Diseases for Oncology, third edition.

Table SII. Patient characteristics overall and by biomarker test.

Characteristic	Overall analysis cohort, n=28,382	MSI (CDx), n=19,894	BRCA 1/2 prostate cancer (CDx), n=106	BRCA 1/2 other cancers (CDx), n=1,422	HRD (CDx), n=295	KRAS/NRAS-RASKET-B (CDx), n=7,232	BRAF-RASKET-B (CDx), n=6,793	KRAS/NRAS-OncoBeam (CDx), n=277	Tissue biopsy (CGP), n=1,55	Liquid biopsy (CGP), n=35
Sex, n (%)										
Male	15,035 (53.0)	10,705 (53.8)	106 (100.0)	256 (18.0)	0 (0.0)	4,398 (60.8)	4,124 (60.7)	172 (62.1)	805 (51.7)	18 (51.4)
Female	13,347 (47.0)	9,189 (46.2)	0 (0.0)	1,166 (82.0)	295 (100.0)	2,834 (39.2)	2,669 (39.3)	105 (37.9)	752 (48.3)	17 (48.6)
Age at index date, mean (SD)	67.5 (11.3)	67.2 (11.0)	72.6 (8.1)	63.5 (11.1)	62.3 (12.4)	71.3 (10.3)	71.2 (10.3)	67.9 (10.3)	61.7 (12.7)	62.7 (14.8)
Body weight at index date										
n/n missing	4,601/23,781	2,882/170	1/105	30/1,392	7/288	2,035/519	1,833/496	12/265	5/1,552	0/35
Mean (SD)	56.9 (13.2)	56.8 (12.9)	52 (-)	53.1 (10.0)	60.4 (5.9)	57.7 (13.6)	58.0 (14.0)	55.5 (8.78)	63.4 (17.9)	NA
Index year, n (%)										
2018	75 (0.3)	75 (0.4)	0 (0.0)	0 (0.0)	0 (0.0)	0 (0.0)	0 (0.0)	0 (0.0)	0 (0.0)	0 (0.0)
2019	7,198 (25.4)	6,882 (34.6)	0 (0.0)	220 (15.5)	0 (0.0)	0 (0.0)	0 (0.0)	0 (0.0)	98 (6.3)	0 (0.0)
2020	8,878 (31.3)	5,781 (29.1)	0 (0.0)	386 (27.1)	0 (0.0)	2,606 (36.0)	2,378 (35.0)	74 (26.7)	592 (38.0)	0 (0.0)
2021	10,088 (35.5)	5,670 (28.5)	79 (74.5)	701 (49.3)	234 (79.3)	3,680 (50.9)	3,510 (51.7)	160 (57.8)	867 (55.7)	35 (100.0)
2022	2,143 (7.6)	1,486 (7.5)	27 (25.5)	115 (8.1)	61 (20.7)	946 (13.1)	905 (13.3)	43 (15.5)	0 (0.0)	0 (0.0)
Total follow-up time in the database, days										
Mean (SD)	349.8 (263.9)	345.3 (280.9)	209.0 (121.6)	413.5 (270.1)	249.7 (119.6)	336.4 (210.8)	334.1 (209.7)	282.9 (187.7)	352.5 (184.9)	232.5 (46.7)
Median (Q1, Q3)	283 (142, 498)	262 (126, 493)	203 (117, 310)	366 (197, 579)	236 (158, 347)	308 (156, 490)	301 (156, 487)	243 (126, 441)	308 (212, 447)	246 (196, 267)
Modified Charlson Comorbidity Index scores during look-back period, mean (SD)	5.3 (2.4)	5.5 (2.4)	5.8 (2.0)	4.8 (2.5)	5.1 (2.3)	4.6 (2.5)	4.6 (2.5)	6.3 (2.1)	5.8 (2.2)	6.7 (2.1)
Comorbidities of interest, n (%)										
Congestive heart failure	3,250 (11.5)	2,130 (10.7)	8 (7.6)	113 (8.0)	26 (8.8)	1,115 (15.4)	1,037 (15.3)	26 (9.4)	138 (8.9)	6 (17.1)
Dementia	279 (1.0)	132 (0.7)	0 (0.0)	2 (0.1)	1 (0.3)	165 (2.3)	156 (2.3)	2 (0.7)	2 (0.1)	0 (0.0)

Chronic pulmonary disease	3,889 (13.7)	2791 (14.0)	6 (5.7)	124 (8.7)	21 (7.1)	928 (12.8)	852 (12.5)	54 (19.5)	259 (16.6)	5 (14.3)
Rheumatologic disease	688 (2.4)	447 (2.3)	1 (0.9)	39 (2.7)	13 (4.4)	198 (2.7)	170 (2.5)	2 (0.7)	50 (3.2)	0 (0.0)
Mild liver disease	6,400 (22.6)	4,663 (23.4)	17 (16.0)	303 (21.3)	50 (17.0)	1,402 (19.4)	1,286 (18.9)	62 (22.4)	373 (24.0)	12 (34.3)
Diabetes with chronic complications	1,613 (5.7)	1,078 (5.4)	3 (2.8)	68 (4.8)	8 (2.7)	548 (7.6)	513 (7.6)	16 (5.8)	43 (2.8)	1 (2.9)
Hemiplegia or paraplegia	148 (0.5)	91 (0.5)	1 (0.9)	4 (0.3)	0 (0.0)	51 (0.7)	42 (0.6)	0 (0.0)	13 (0.8)	0 (0.0)
Renal disease	1,118 (3.9)	764 (3.8)	7 (6.6)	26 (1.8)	9 (3.1)	386 (5.3)	358 (5.3)	13 (4.7)	34 (2.2)	2 (5.7)
Any malignancy, including lymphoma and leukemia	27,926 (98.4)	19,732 (99.2)	106 (100.0)	1,420 (99.9)	294 (99.7)	6,900 (95.4)	6,488 (95.5)	277 (100.0)	1,548 (99.4)	35 (100.0)
Moderate or severe liver disease	320 (1.1)	257 (1.3)	0 (0.0)	7 (0.5)	0 (0.0)	66 (0.9)	66 (1.0)	3 (1.1)	11 (0.7)	0 (0.0)
Metastatic solid tumor	16,819 (59.3)	12,676 (63.7)	84 (79.3)	719 (50.6)	176 (59.7)	2,996 (41.4)	2,831 (41.7)	228 (82.3)	1,114 (71.6)	30 (85.7)
HIV	17 (0.1)	10 (0.1)	0 (0.0)	2 (0.1)	0 (0.0)	1 (0.0)	1 (0.0)	0 (0.0)	3 (0.2)	0 (0.0)

Note: A patient may have received more than one biomarker test. CDx: companion diagnostics; CGP: comprehensive genomic profiling; HIV, human immunodeficiency virus; HRD, homologous recombination deficiency; MSI, microsatellite instability; SD, standard deviation; Q1, quartile 1; Q3, quartile 3; NA, not available.
